# Evaluation of the Anti-schistosomal Effects of Turmeric (*Curcuma longa*) Versus Praziquantel in *Schistosoma mansoni* Infected Mice

**Published:** 2017

**Authors:** Atef HUSSEIN, Samia RASHED, Ibrahim El HAYAWAN, Rabab El-SAYED, Hemat ALI

**Affiliations:** Dept. of Parasitology, Faculty of Medicine, Benha University, Benha, Egypt

**Keywords:** Turmeric, *Schistosoma mansoni*, Praziquantel

## Abstract

**Background:**

Curcumin is the major active ingredient of *Curcuma longa L.*, traditionally known as turmeric and has been shown to exhibit a wide range of pharmacological activities including anti-parasitic effect. However, it is found to be water-insoluble and has low bioavailability. The aim of this study was to explore the potential role of turmeric solved in olive oil either alone or in combination with praziquantel (PZQ) in treatment of schistosomiasis *mansoni*.

**Methods:**

The whole turmeric powder suspended in olive oil (as a solvent) is indicated to *S. mansoni*-infected mice aiming to study its potential therapeutic role, either alone or in combination with PZQ.

**Results:**

Turmeric significantly reduced *S. mansoni* worm burden and complete absence of adult worms achieved in mice treated with combination of turmeric and PZQ. Turmeric has slight non-significant effect on the oogram pattern in all examined *S. mansoni* infected mice. Turmeric and PZQ found to exert a significant reduction of granuloma size in comparison with control. However, turmeric has a non-significant effect on granuloma number. On the other hand, turmeric or/and PZQ treated mice showed obvious improvement of pathology with mild cloudy swelling and less hydropic degeneration.

**Conclusion:**

Turmeric significantly reduced parasite worm burden, granuloma size and consequently the pathology of affected liver, it still far less effective than PZQ.

## Introduction

Schistosomiasis remains one of the most prevalent helminthic infections in the world. The number of active schistosome infections was more likely between 391 and 587 million people worldwide, with a global disease burden calculated at 24–56 million disability-adjusted life-years lost (1). As there is no effective vaccine against schistosomiasis, current treatment relies on a single drug, praziquantel (PZQ). With increased efforts to control this disease by mass treatment, the possibility of PZQ resistance developing is a serious concern (2). Thus, there is a need for sustained research toward developing alternative chemotherapeutic compounds against schistosomiasis. Plants are possible sources of novel drugs and many plants have been investigated for possible antiparasitic effects (3–5).

Turmeric, the rhizome of *Curcuma longa L.* (*C. longa*) has been used since ancient times as a spice, coloring, flavoring, and traditional medicine. Curcumin [1,7-bis (4-hydroxy-3-methoxyphenyl) -1,6- heptadiene -3, 5-dione] is the major active ingredient of turmeric and has been shown to exhibit a wide range of pharmacological activities including anti-inflammatory, anti-cancer, anti-oxidant, anti-atherosclerotic, anti-microbial and wound healing effects (6). Moreover, it has antiparasitic effect against some protozoal infections as *Trypanosoma brucei* (7), *Giardia lamblia* (8) and *Leishmania donovani* (9). On that basis, the schistosomicidal and the immunomodulatory effects of oil extract of turmeric against *Schistosoma mansoni* infected mice had been investigated (10–14).

Despite curcumin being a powerful bioactive agent and natural antioxidant, it is found to be water-insoluble and has low bioavailability (15), a character that may minimize its therapeutic value. Olive oil and non-curcuminoid components of turmeric reported to increase the solubility and bioavailability of curcumin (16, 17). The aim of this study was to explore the potential role of turmeric solved in olive oil either alone or in combination with PZQ in treatment of schistosomiasis *mansoni*.

## Materials and Methods

### Herbal Extract

Turmeric powdered material, obtained from local conventional herbal medicine market, suspended in olive oil as a solvent reagent at a concentration of 80 mg/ml.

### Parasites and animals

Cercariae of *S. mansoni* were obtained from infected *Biomphalaria alexandrina* snails, reared and maintained at Schistosome Biological Supply Program (SBSP), Theodor Bilharz Research Institute, Giza, Egypt. Eighty laboratory-bred male Swiss albino mice, CD1 bred, were used in this study. All mice were infected with 50 *S. mansoni* cercariae suspended in 0.2 ml water via subcutaneous injection.

### Experimental design

This study was conducted during the period between April 2015 and January 2016. Infected mice were divided into 4 batches, 20 mice each, representing Cur treated group, PZQ treated group, Turmeric/PZQ treated group and untreated control group. Both turmeric treated groups were further divide into 2 subgroups (one group treated at 1^st^ week post-infection and the second treated at the 4^th^ weak post-infection). Turmeric dose given was 400 mg/kg body weight, twice/week for 8 consecutive weeks, using a stainless steel oral cannula. The dose was modified from that used by El-Ansary and her colleagues (10). At six weeks post-infection, PZQ (Alexandria Company for Pharmaceuticals and Chemical Industries, Alex., Egypt) was freshly suspended in 13 ml of 2% cremophore-EL) Sigma Chemical Co., USA) and orally administered to mice at a dose given to 500 mg/kg body weight for two consecutive days (18).

### Evaluation of turmeric schistosomicidal effect

#### A. Worm Burden

Adult schistosome recovery assessed by porto-mesenteric perfusion technique, two weeks post-treatment, according to the method of Duvall and DeWitt (19).

#### B. Oogram pattern

After mice perfusion, three fragments, one cm in length of the small intestine were cut longitudinally, rinsed in saline, slightly dried on filter paper, compressed between two glass slides and examined under microscope for oogram pattern that may reflect presence of direct drug action on ova development (20). We classified the eggs of *S. mansoni* in three type’s immature, mature and dead ones (21).

#### C. Histopathological examination

Mice livers were fixed for 48 h in 10% buffered formalin and then embedded in paraffin. Five sections (5 microns in thickness) were taken from each liver specimen, each section being at a distance of at least 500 μm from the preceding one (22). Sections were stained with hematoxylin and eosin (23) for granuloma counting and Masson trichrome stains (24) for demonstration of collagen fibers. Lesions containing single ova in their centers were selected for measurement (25). First, the greatest diameter of the lesion was obtained, then the ocular micrometer was rotated 90 degrees, and the diameter perpendicular to the first one was measured (22). The size of each liver granuloma was calculated from the mean diameter of each lesion on the assumption that they were spherical (26). For each section, granuloma is counted in five successive fields (10×10).

### Statistical analysis

The data were recorded on an “Investigation report form”. These data were tabulated, coded then analyzed using the computer program SPSS version 16 (Chicago, IL, USA). ANOVA (analysis of variance) test used to compare between more than two groups of numerical (parametric) data and Kruskal Wallis test between more than two groups of numerical (non parametric) data. Student’s t-test used to compare between mean of two groups of numerical (parametric) data. For continuous non-parametric data, Mann-Whitney U-test used for inter-group analysis. *P* value <0.05 was considered statistically significant and a *P* value <0.0001 was considered highly significant in all analyses.

### Ethical considerations

The study was approved by Parasitology Department Research Committee and the Ethical Committee at the Faculty of Medicine, Benha University.

## Results

**Fig. 1:**
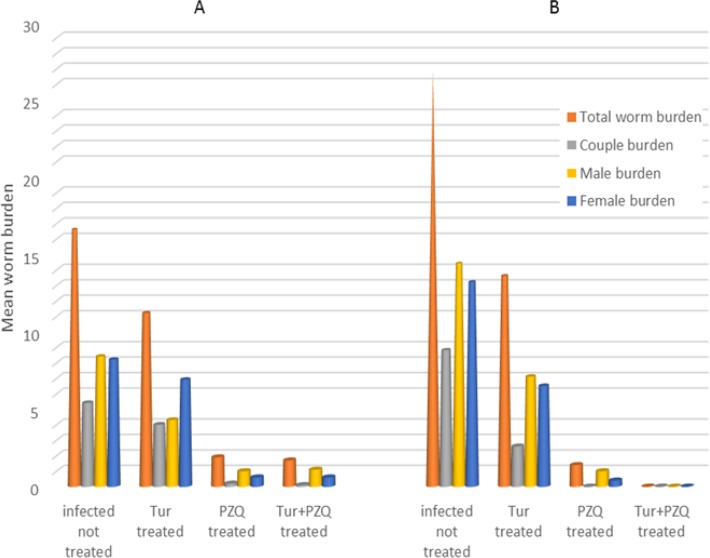
Effect of turmeric and PZQ on *S. mansoni* worm burden in mice. **A:** Turmeric dose = 400 mg/kg body weight, twice/week for 8 consecutive weeks, starting one week postinfection (pi). PZQ dose = 500 mg/kg body weight for two consecutive days, starting 6 wk pi. Animals were examined at week 8 pi. **B:** the same as above, but turmeric started 4 wk pi and the animal examined 12 wk pi.

Turmeric significantly reduced both male and female *S. mansoni* worm burden whether given one weak (49.3%) or 4 wk (38.4%) post-infection. A high significant schistosomicidal effect of PZQ noticed in all groups, either as a monotherapy or in combination with Turmeric. The highest effect was on couple burden counted 12 wk post infection (100%). Female worms were more sensitive (96.2%) to PZQ than males (91.2%). Complete absence of adult worms achieved in mice treated with combination of turmeric and PZQ and sacrificed 12 wk post infection (Table 1, Fig. 1).

**Table 1:** Effect of turmeric and PZQ on *S. mansoni* worm burden in infected mice

***Experimental groups***	***Treatment after infection (weeks)***	***Examined (weeks)***	***Mean worm burden (%reduction)***						
			**Total worm**	**Couple**	**Male**	**Female**
Infected not treated	-	8	16.6	5.4	8.4	8.2
	-	12	26.8	8.8	14.4	13.2
Turmeric treated^a^	1	8	11.2(33%)^*^	4.0(26%)	4.3(49%)^**^	6.9(16%)
	4	12	13.6(49%)^**^	2.6(71%)^**^	7.1(51%)	6.5(50%)^**^
PZQ treated^b^	6	8	1.6(90%)^**^	0.2(96%)^**^	1.0(88%)^**^	0.6(93%)^**^
		12	1.4(95%)^**^	0.0(100%)	1.0(93%)^**^	0.4(97%)^**^
Turmeric + PZQ treated	1 (Cur) + 6(PZQ) 4 (Cur) + 6 (PZQ)	8	1.7 (90%)^**^	0.1(98%)^**^	1.1(87%)^**^	0.6(93%)^**^
		12	0.0(100%)	0.0(100%)	0.0(100%)	0.0(100%)

a Turmeric dose = 400 mg/kg body weight, twice/week for 8 consecutive weeks.

b PZQ dose = 500 mg/kg body weight for two consecutive days.

* *P*<0.05:Significant difference between treated groups versus control group.

** *P*<0.001: Significant difference between treated groups versus control group.

**Fig. 2:**
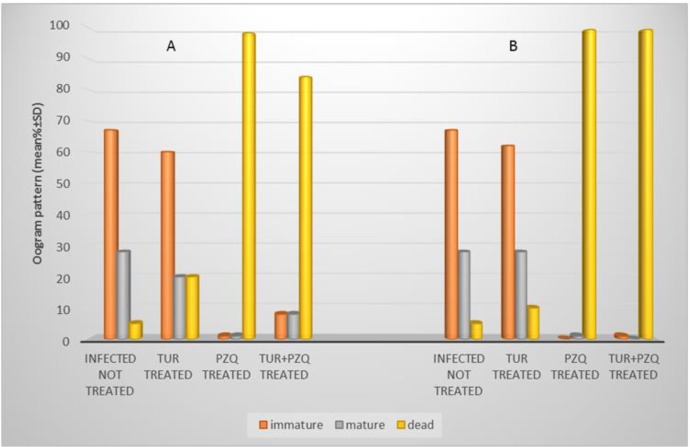
Effect of turmeric and PZQ on oogram pattern in intestinal segments of *S. mansoni* infected mice. **A**. Turmeric dose =400 mg/kg body weight, twice/week for 8 consecutive weeks, starting one-week pi. PZQ dose =500 mg/kg body weight for two consecutive days, starting 6 wk pi. Animals were examined at week 8 pi. **B**. The same as above, but turmeric started 4 wk pi and the animal examined 12 wk pi.

Turmeric has slight non-significant effect on the oogram pattern in all examined *S. mansoni* infected mice. While PZQ exert a highly significant increase in the percentage of dead ova when it compared with control untreated groups. Concerning combined therapy effect, there was complete disappearance of mature ova and highly significant increase in the percentage of dead ova (Table 2, Fig. 2).

**Table 2:** Effect of turmeric and PZQ on oogram pattern in intestinal segments of *S. mansoni* infected mice

***Experimental groups***	***Treatment after infection (weeks)***	***Examined (weeks)***	***Oogram pattern (mean %± SD)***		
			**immature**	**mature**	**dead**
Infected not treated	-	8	67±7.9	28±5.1	5±6.9
	-	12	67±7.9	28±5.1	5±6.9
Cur treated^a^	1	8	60±6.4	20±5.7	20±9.1
	4	12	62±12.4	28±11.4	10±9.9
PZQ treated^b^	6	8	1.0±0.4^**^	1±0.7^**^	98±0.5^**^
		12	0.0	1±0^**^	98±0.5^**^
Cur + PZQ treated	1 (Cur) + 6(PZQ)	8	8±2.7^**^	8±4.6^**^	84±6.3^**^
	4 (Cur) + 6 (PZQ)	12	1.0±0^**^	0	99±0.5^**^

a Cur dose = 400 mg/kg body weight, twice/week for 8 consecutive weeks.

b PZQ dose = 500 mg/kg body weight for two consecutive days.

** *P*<0.001: Significant difference between treated groups versus control group.

There is moderate reduction in granuloma number in turmeric treated group when compared with control, yet statistically non-significant.

**Fig. 3:**
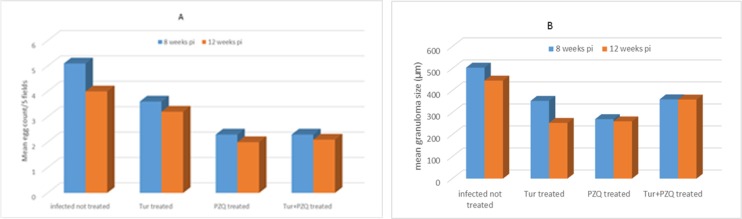
Effect of Cur and PZQ treatment on hepatic granuloma formation in *S. mansoni* infected mice. **A.** Turmeric dose =400 mg/kg body weight, twice/week for 8 consecutive weeks, starting one week pi. PZQ dose =500 mg/kg body weight for two consecutive days, starting 6 wk pi. Animals were examined at week 8 pi. **B.** the same as above, but turmeric started 4 wk pi and the animal examined 12 wk pi.

However, PZQ and Turmeric/PZQ treatment resulted in statistically significant decrease in granuloma number (54.9%). Turmeric and PZQ found to exert a significant reduction of granuloma size in comparison with control (Table 3, Fig. 3).

**Table 3:** Effect of Cur and PZQ treatment on hepatic granuloma formation in *S. mansoni* infected mice

***Experimental groups***	***Treatment after infection (weeks)***	***Examined (weeks)***	***Hepatic granuloma***			
			**Mean no/5 fields±SD (reduction%)**	**Mean size (μm)±SD (reduction%)**	**% Cellular granuloma**	**%Fibrocellular granuloma**
Infected not treated	-	8	5.1±1.3	445±60	10%	90%
	-	12	4.0±1.6	504±37	9%	91%
Cur treated^a^	1	8	3.6±0.96 (29.4%)	253±39 (43.1%)^*^	9%	91%
	4	12	3.2±1.2 (20%)	353±29 (30%)^*^	8%	92
PZQ treated^b^	6	8	2.3±0.7 (54.9%)^*^	260±35 (42.6%)^*^	23%	77%
		12	2.0±0.7 (50%)^*^	270±35 (46.4%)^**^	18%	82%
Cur + PZQ	1(Cur) + 6(PZQ)	8	2.3±0.9 (54.9%)^*^	359±44 (19.3%)^*^	18%	82%
	4(Cur) + 6(PZQ)	12	2.1±0.7 (47.5)^*^	360±15 (28.6%)^*^	8%	92%

a Cur dose = 400 mg/kg body weight, twice/week for 8 consecutive weeks

b PZQ dose = 500 mg/kg body weight for two consecutive days

* *P*<0.05:Significant difference between treated groups versus control group

** *P*<0.001: Significant difference between treated groups versus control group

Treatment of the *S. mansoni* infected mice did not show any statistically significant change in the frequency pattern of the cellular and fibrocellular granuloma (Table 3) in all studied mice groups.

In infected untreated mice, histopathological examination of liver sections shows apparent disruption of the lobular architecture with many granulomas in the hepatic parenchyma and to a lesser extent the portal tracts. The liver cells show cloudy swelling with moderate hydropic degeneration of hepatocytes. Necrosis is constantly present at sites of granuloma. Liver parenchyma showed dense inflammatory cells infiltration and dilatation of blood sinusoids.

On the other hand, turmeric or/and PZQ treated mice showed obvious improvement of pathology with mild cloudy swelling and less hydropic degeneration.

## Discussion

Curcumin is the major active component of turmeric (6). In spite of its efficacy and safety, curcumin has not yet been approved as a therapeutic agent in part perhaps due to the poor aqueous solubility and relatively low bioavailability (15,27). “Different methods have been performed to increase the absorption of curcumin including nanocrystals, emulsions, liposomes, self-assemblies and nanogels” (28). A combination of curcumin with the noncurcuminoid components of turmeric has been resulted in 6.93 folds increase in curcumin bioavailability (17). Besides, the use of Olive oil as a solvent is reported to increase the solubility and bioavailability of turmeric (16).

In the current study, commercial turmeric solved in olive oil was used aiming to study its potential role either alone or in combination with PZQ in treatment of *S. mansoni* infection. The anti-schistosomicidal effect of curcumin has been studied in few researches, to the best of our knowledge; only three reported the reducing effect of curcumin on worm burden using methanol extract of *C. longa* (10, 13, 29). Turmeric caused significant reduction in the *S. mansoni* female and male worm burden. Moreover, a high significant reduction of worm couples noticed. A 67.3% reduction was reported in adult Schistosoma’s worm burden in mice, using a single dose of curcumin (20 mg) (29). More or less similar results that used methanol extract of *C. longa,* were reported a schistosomes worm burden reduction of 55% and 44.4%, respectively (10, 13). In contradiction to these reports, an oral dose of curcumin was reported for two months before infection, had no effect on the total worm burden (30). When administered orally, curcumin reached the maximum concentration at 1 h, and declined to below the detection limit within 6 hours (31). Our data showed increase number of recovered worms in untreated control group, examined after 12 wk post infection. “This could be related to the well-established biological fact that the time required by the immature schistosome worms to reach the portal-hepatic system, coming from the lungs is variable” (32). In addition, the noticed increase worm burden reduction, in mice treated up to 12 wk post-infection when compared with mice treated until the 8th wk post-infection, could allow us to assume that turmeric is more effective against adult *S. mansoni* than juvenile stages. Our results showed that PZQ is still significantly very effective as an anti-schistosomal drug in comparison with control-infected group. This effect enhanced by combination of PZQ and Turmeric, as evident by complete absence of the adult worms from infected mice when compared with mice treated by monotherapy.

The study of the oogram pattern for enumeration of the various egg types is an easy and reliable method of evaluating the therapeutic value of anti-schistosomal drugs (32). In the current study, turmeric failed to prove any significant effect on oogram pattern compared to control, which denotes that curcumin has no effect on the oviposition of the viable adult worm. These results are in parallel with that reported by a previous study, which showed that curcumin has no effect on the adult fecundity (13). Conversely, PZQ caused a significant increase in the percentage of dead eggs and significant reduction in the percentage of viable eggs when compared with their corresponding infected untreated groups. These results are in agreement with previous studies, which reported reduction in the number of mature ova, an increase in the dead ova counts and a disappearance of immature eggs stages in mice infected with *S. mansoni* and treated with PZQ (33–37). Liver granuloma response around schistosome eggs is a hallmark of *S. mansoni* infection (38). Treatment with successful schistosomicidal drugs eradicates the worms and stopping their egg production. At the same time, these drugs may modulate the host reaction to eggs (39).

In the current study, turmeric treated group shows notable reduction in granuloma number (*P*>0.05) and size (*P*<0.05) in comparison with the control group. While PZQ treated groups show a significant decrease in number and size of hepatic granuloma (*P*<0.05). These results are in agreement with those of other researchers, who reported reduction of granuloma size after administration of either curcumin (11, 13) or PZQ (40). Curcumin might modulate granuloma formation through regulation of cytokines expression, especially TNF- α (13). Moreover, it has found to inhibit the release of both IL-2 (41) and IL-4 (42) that reported to play a role in granuloma formation (43). Concerning the combination of both turmeric and PZQ, our results showed unexplained lower effect on granuloma number and size than that noticed for monotherapy with PZQ. This may be due to different effects of both drugs on the interleukins as PZQ increases TNF-α, IL-6, IL-8, IFN-γ, IL-12p70, and IL-23 levels, (44) while turmeric suppress TNF-α, IL-6 and IL-8 (45).

In the current study, the granulomatous lesions were mostly seen in the hepatic parenchyma and to a lesser extent in the portal tracts of all infected mice with fibrocellular granuloma constituted more than 82% of the estimated granulomas. Fibrocellular granulomas, were constituted 60%–100% of the estimated granulomas (46, 47). Histopathological examination of liver revealed presence of areas of necrosis in 2nd and 3rd months post infection. These results agree with those obtained by EL assar et al (48). Regarding its histopathological effect on liver parenchyma and hepatocytes, turmeric therapy ameliorated the condition of liver tissue affection due to *S. mansoni* infection. Turmeric reduced the cloudy swelling and hydropic degeneration of hepatocytes as compared by the control infected non-treated mice. This improvement may be due to the anti-inflammatory effect of turmeric (6). Similar result was noticed in PZQ treated mice. This comes in agreement with the study of Gerges, (49) who observed improvement of the hepatocytes, which became healthier after PZQ therapy.

The small number of experimental groups limits our result. A larger-scale study is necessary to emphasize the efficiency of turmeric as anti-schistosomal drug.

## Conclusion

Turmeric significantly reduced parasite worm burden, granuloma size, number and pathology of affected liver. However, its effect is much less effective than that of the traditionally applied drug, PZQ. The association of turmeric and PZQ does not significantly enhance their anti-schistosomal activity
